# Highly Flexible and Conformable ZnO/FeGa Magnetoelectric Heterostructures for Skin wound Healing

**DOI:** 10.1002/advs.202523781

**Published:** 2026-06-12

**Authors:** Filippos Perdikos, Laia Bagur, Cristina Vaca, Aritz Lafuente, Joaquin Llacer‐Wintle, Bojan Ambrožič, Christina Stefani, Minsoo Kim, Goran Dražić, Darja Lisjak, Jordi Sort, Xiang‐Zhong Chen, Maria Jose Esplandiu, Salvador Pané, Carme Nogués, Josep Nogués, Andreu Blanquer, Borja Sepúlveda

**Affiliations:** ^1^ Catalan Institute of Nanoscience and Nanotechnology (ICN2) CSIC and BIST Bellaterra Barcelona Spain; ^2^ Departament de Biologia Cel·lular, Fisiologia i Immunologia Universitat Autònoma de Barcelona Bellaterra Spain; ^3^ Instituto de Microelectronica de Barcelona (IMB‐CNM, CSIC) Bellaterra Barcelona Spain; ^4^ Multi‐Scale Robotics Lab (MSRL) Institute of Robotics and Intelligent Systems (IRIS) Zürich Switzerland; ^5^ Center Odličnosti Nanoznanosti in Nanotehnologije Ljubljana Slovenia; ^6^ Departament de Física Universitat Autònoma de Barcelona Bellaterra Spain; ^7^ National Institute of Chemistry Ljubljana Slovenia; ^8^ Department for Materials Synthesis Jožef Stefan Institute Ljubljana Slovenia; ^9^ ICREA Bracelona Spain; ^10^ International Institute for Intelligent Nanorobots and Nanosystems College of Intelligent Robotics and Advanced Manufacturing State Key Laboratory of Photovoltaic Science and Technology Shanghai Frontiers Science Research Base of Intelligent Optoelectronics and Perception Institute of Optoelectronics Fudan University Shanghai China; ^11^ Zhejiang Key Laboratory of Extreme Environment Functional Materials Yiwu Research Institute of Fudan university Yiwu China

**Keywords:** FeGa, flexible magnetoelectrics, magnetoelectric coupling, magnetostriction, piezoelectric, skin cells stimulation, wound healing, ZnO

## Abstract

Composite magnetoelectric (piezoelectric/magnetostrictive) materials are gaining an increased interest due to their appealing wireless actuation capabilities. For many applications, such as sensing, energy harvesting or cell electrostimulation, flexible and adaptable heterostructures are required. However, some of the proposed structures suffer from poor magnetoelectric performance due to their inherent stiffness and the resulting substrate clamping effects. Here, a highly flexible and conformable nanostructured ZnO(piezoelectric)/FeGa(magnetostrictive) magnetoelectric heterostructure embedded in a low Young's modulus elastomer (polydimethylsiloxane; PDMS) is presented. This heterostructure is designed to enhance wound healing via electric stimulation induced by low‐intensity and low‐frequency AC magnetic fields. In vitro testing using normal human dermal fibroblasts (NHDF) and human keratinocytes (HaCaT) cell cultures demonstrated high cell viability and no adverse effects under magnetoelectric stimulation. Moreover, the magnetoelectric stimulation significantly enhanced keratinocyte stratification and fibroblast collagen production, with remarkable improvements in cell migration compared to non‐stimulated cells. These results underscore the potential of highly flexible magnetoelectric heterostructures as active dressings to improve wound healing processes, especially relevant for chronic wounds and other epithelial disorders.

## Graphical abstract

The magnetic field‐induced electric field generated by a highly flexile ZnO(piezoelectric)/FeGa(magnetostrictive) magnetoelectric heterostructure embedded in the low Young's modulus elastomer PDMS has been used to stimulate the wound healing processes.

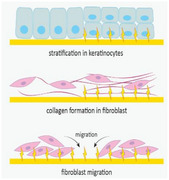

## 1 Introduction

Magnetoelectric materials are structures in which the magnetic and electrical properties are coupled to each other. This coupling allows the electric polarization to be tuned by magnetic fields or the magnetization by electric fields, making these materials appealing for a wide range of technological areas [1, 2, 3, 4]. Importantly, many applications, such as in biomedicine, [5, 6, 7] sensing, [8, 9, 10, 11, 12] soft robotics [13] and energy harvesting, [10, 14, 15] require these materials to be elastic and conformable. Flexible heterostructures also minimize clamping effects from the use of rigid substrates, which reduce strain‐mediated magnetoelectric coupling. Thus, in recent years there has been considerable effort toward the development of flexible composite magnetoelectric (magnetostrictive/piezoelectric) heterostructures [16, 17, 18]. In these materials, when a magnetic field is applied, the magnetostrictive component suffers a mechanical deformation. This mechanical strain is transferred to the adjacent piezoelectric structure, which generates an electric field [1]. To develop flexible magnetoelectric structures, two common approaches can be found: [16, 17] (i) growing or embedding the magnetoelectric composites in flexible polymer‐based or inorganic substrates (e.g., polyurethane, [19] mica, [20] polydimethylsiloxane, [12, 13, 21] Kapton, [22] or polyethylene terephthalate [23, 24]), or (ii) using flexible piezoelectric polymers, such as polyvinylidene fluoride‐based polymers [25, 26]. Despite their potential properties, in most cases both types of approaches exhibit still moderate stiffness originating from the relatively high Young's modulus of the flexible layers or the overall structure, which may hinder their magnetoelectric properties. Therefore, highly flexible magnetoelectric systems could enable stronger magnetoelectric effects and innovative applications exploiting their integration into different devices requiring highly flexible and conformable structures. This could be especially relevant in biomedical applications, such as wireless skin stimulation to enhance wound healing processes.

Wound healing is a complex process that requires the coordination of multiple physiological mechanisms working simultaneously in the restoration of the injured area and the recovery of the functionality of the epithelial tissue. However, in some cases, these physiological processes can be inefficient, resulting in chronic wounds, especially in individuals with risk factors, such as advanced age, malnutrition, or underlying conditions like cancer or diabetes [27]. In addition, infections frequently occur in chronic wounds, and one of the primary causes of treatment failure is the development of biofilms. Biofilms are structured microbial communities embedded within an extracellular polymeric substance secreted by pathogenic bacteria, and they are present in more than 70% of chronic wounds [28]. Although several strategies, including debridement, hydrogels, and light‐based therapies, have been proposed to reduce biofilm formation, complete eradication is rarely achieved [29]. Additional approaches under investigation include aptamers, antimicrobial peptides, and nanotechnology‐based therapies [30]. Metal ions at low concentrations, as well as metallic nanoparticles such as silver and zinc, have also shown effectiveness against pathogenic bacteria and biofilm formation [31, 32, 33].

The wound healing processes can be stimulated by electric fields and currents. Under normal conditions, the skin maintains a transepithelial potential of 50 mV. When an injury occurs, the wound site becomes electrically negative compared to the undamaged regions, forming a current density of 1 to 10 µA/cm^2^ in the damaged region and up to 300 µA/cm^2^ at the edge of the wound. These currents generate an electric field ranging from 100 to 150 mV/mm, decreasing to zero upon complete repair. This electric field, along with biochemical cues, is one of the signals that initiate the healing process [27, 34]. Interestingly, the development of novel conductive materials together with advanced power‐supply systems has expanded the possibilities for delivering controlled electrical stimulation for wound healing and biofilm eradication. Notable examples are conductive membranes, such as polypyrrole combined with polylactic acid, [35] as well as hydrogels enriched with conductive materials [36, 37]. Additionally, flexible photovoltaic electrodes and triboelectric nanogenerators have been studied for electrical stimulation therapies [38]. Although the application of external electric fields appears to be a promising approach for skin regeneration, [39] its clinical adoption presents challenges due to the invasive nature of the electrodes and the need for toxic batteries.

In response to these issues, piezoelectric materials have emerged as a viable alternative, since these materials can generate electric fields when subjected to mechanical stimuli. This property allows for electrical stimulation without the need for electrodes and batteries. Consequently, several studies have already demonstrated their potential for biomedical applications, highlighting their promise in the field of tissue regeneration and repair [1, 40, 41, 42, 43, 44, 45, 46, 47, 48, 49]. Usually, the wireless mechanical stimulation of piezoelectric materials for biomedical applications is carried out using ultrasounds [40, 41, 42, 43, 44]. However, ultrasounds may have adverse effects on the human body [50, 51] and have a frequency that is several orders of magnitude higher than typical cellular processes, which are in the millisecond range scale [22, 52]. Thus, in recent years alternative approaches are being investigated. In particular, magnetoelectric materials have demonstrated their potential in diverse biomedical applications, such as bone repair, nerve regeneration or muscle stimulation [1, 5, 45, 46, 53, 54, 55, 56, 57]. However, the use of magnetoelectric heterostructures in wound healing has been explored only to a limited extent [46, 58].

**SCHEME 1:**
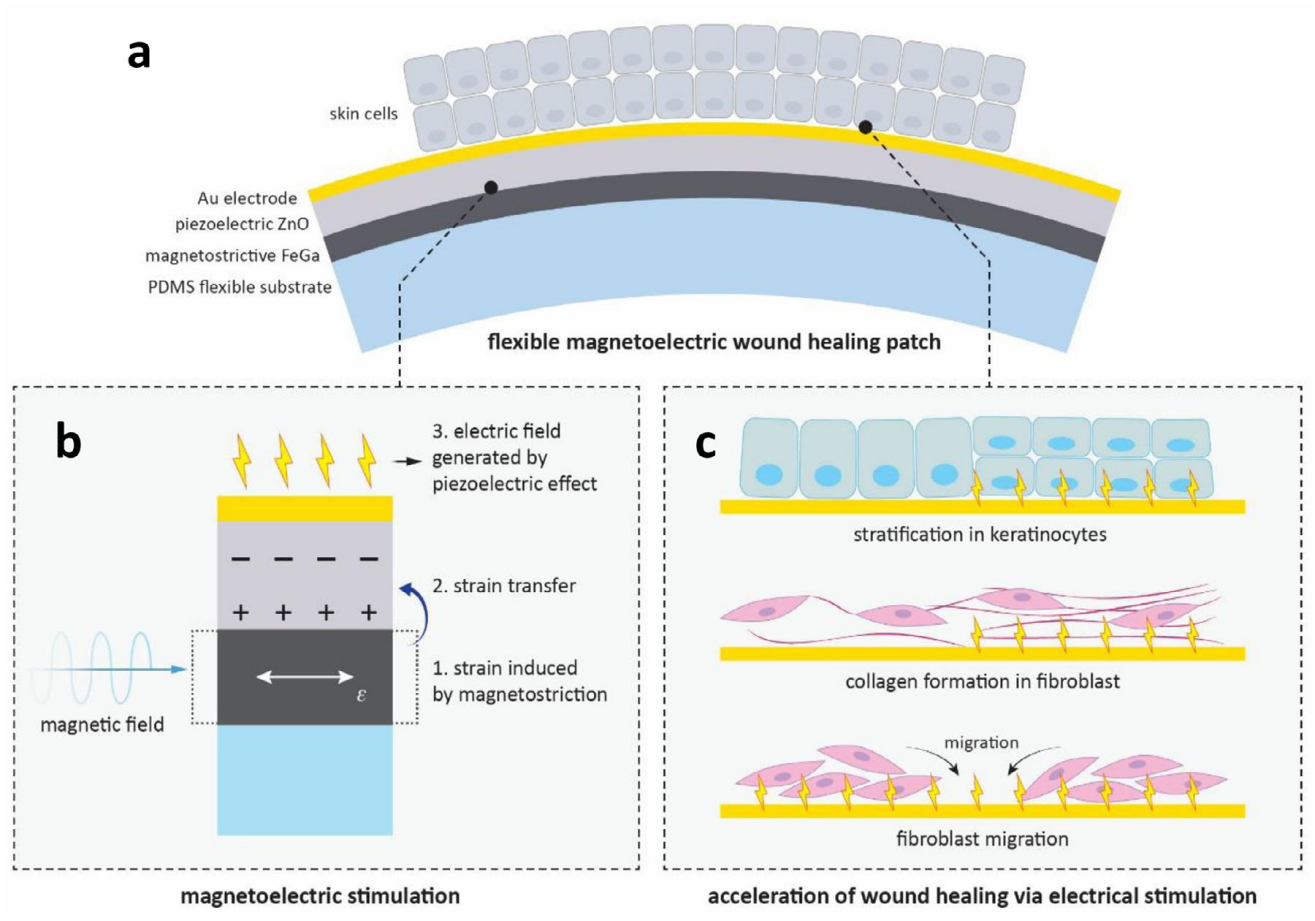
a) Schematic representation of the piezoelectric/magnetostrictive structure. b) Working principle of the electric field generation of piezoelectric/magnetostrictive heterostructure by the applied magnetic field. c) Different effects of the induced electric fields on the wound.

Here, a highly flexible magnetoelectric heterostructure composed of hydrothermally synthesized piezoelectric ZnO nanosheets covered with a magnetostrictive FeGa layer and embedded in low Young's modulus polydimethylsiloxane (PDMS) elastomer layer has been developed. This heterostructure, when subjected to low intensity and low frequency magnetic actuation, generates an electric stimulation that enables enhancing wound healing processes by promoting key skin cell activities, such as cell proliferation, differentiation and migration, as summarized in Scheme 1.

## 2 Results and Discussion

### 2.1 Fabrication of the Magnetoelectric Heterostructures

**FIGURE 1:**
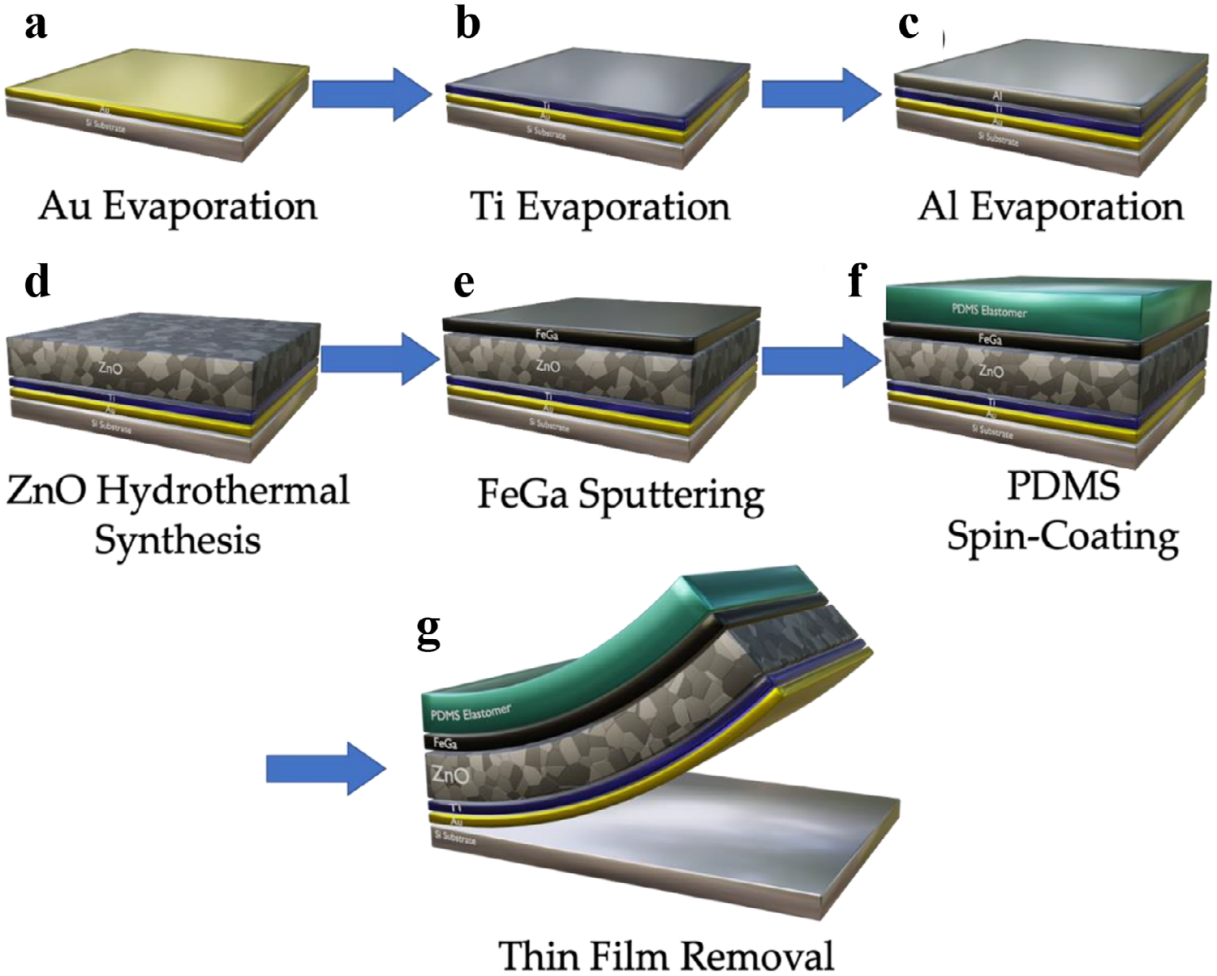
Schematic description of the fabrication process of the FeGa/ZnO heterostructure integrated in the PDMS elastomer. See also Figure S1.

The magnetoelectric heterostructure is composed of Au (50 nm) / Ti (10 nm) (electrode/intermediate layer) / ZnO (8.5 µm) (piezoelectric) / FeGa (100 nm) (magnetostrictive) films integrated in a flexible PDMS (52 µm) elastomer layer (Figure 1 and Figure S1). While ZnO possesses only a moderate piezoelectric coefficient (typically in the 10–20 pm/V range), it offers the significant advantages of being inexpensive, readily fabricated with high crystallinity at low temperature, and scalable for industrial production. The FeGa offers a large magnetostrictive coefficient (typically in the range 300–400 × 10^−6^ for Fe‐rich alloys) and can be deposited with high conformality in large areas by sputtering. Finally, the PDMS was selected due to its low Young's modulus, biocompatibility and capacity to penetrate inside nanostructured layers in the uncured state.

The fabrication process was designed and optimized to enable a straightforward transfer of the grown ZnO/FeGa nanostructured layer into the flexible PDMS films, and to provide a chemically resistant and biocompatible Au electrode to be in contact with the cells.

The fabrication of the heterostructures (Figure 1 and Figure S1) began with the electron beam deposition of the Au (50 nm), Ti (10 nm) and Al (100 nm) trilayer on [100]‐oriented Si substrates (Figure 1a–c). The role of the Au layer was to serve as an electrode, but also as a low adhesion layer with the silicon substrate to enable the subsequent transfer of the magnetoelectric heterostructure to the flexible PDMS. Its thickness was selected to provide a robust and continuous electrode film after the transfer. The 10 nm Ti intermediate layer was necessary to fully cover the Au layer and prevent the catalytic growth of ZnO nanostructures on the Au surface, which could hamper the robust transfer of the magnetoelectric heterostructure. The 100 nm Al layer, which is dissolved during the ZnO growth process, was necessary to catalyze the synthesis of ZnO with nanosheet shape and to achieve high Al doping concentration to maximize the chemical stability of the nanosheets.

For the synthesis of the ZnO nanosheets (Figure 1d), an equimolar (5 mm) aqueous solution of water‐soluble zinc nitrate hexahydrate (Zn(NO)_3_)_2_·6H_2_O) and basic buffer hexamethylenetetramine (HMTA) was magnetically stirred and then sonicated for 10 min. The Si/Au/Ti/Al multilayer was placed upside down on the solution, floating at the water/air interface to enable the growth of ZnO nanosheets only at the metal interface. The solution with the floating sample was hermetically sealed to avoid water evaporation and placed in an oven for 14 h at 80°C for the hydrothermal growth to achieve a homogeneous ZnO nanosheets surface coverage. During the synthesis, the Zn(NO_3_)_2_·6H_2_O provided Zn^2+^ ions, while the HMTA created an alkaline environment, [59] in which the Zn^2+^ ions reacted with OH^−^ ions to form zinc hydroxide. The zinc hydroxide then underwent dehydration and recrystallization to form the ZnO nanostructures [59, 60, 61]. During the growth process, the Al layer was dissolved and was partially incorporated into the ZnO nanostructures, thus promoting the formation of the Al‐doped ZnO nanosheets [62, 63]. After the reaction, the sample was transferred to ethanol for the drying process to avoid the strong water capillary forces that can damage the piezoelectric ZnO nanosheets [64]. This growth conditions provided an optimized robust and flexible architecture. Regarding the underlying ZnO architecture, its density and morphology were already systematically optimized, [65] where at low ZnO concentrations, the isolated ZnO nanosheets are excessively fragile and prone to capillary‐driven collapse during the drying process. Conversely, at higher concentrations, the dense packing compromises the subsequent sputtering coverage of the FeGa layer. Moreover, an over‐concentrated ZnO network becomes highly interconnected and rigid, which negatively affects the mechanical flexibility of the final heterostructure when transferred onto the elastic PDMS substrate. Thus, the ZnO thickness used in this study was a compromise between the fragility of the low ZnO concentration and the excessive density and the stiffness of the high ZnO concentrations.

Next, a Fe_80_Ga_20_ layer with a nominal thickness of 100 nm (on a flat surface) was sputtered on the ZnO nanosheets to generate the magnetostrictive actuation (Figure 1e; see Experimental Section). The FeGa alloy composition was selected to maximize the magnetostrictive effects, and the nominal thickness to achieve optimized magnetic induced mechanical actuation on the ZnO nanosheets, as demonstrated in the following section.

To systematically analyze the magnetically induced mechanical actuation of the FeGa on the ZnO nanosheets, we compared the magnetoelastic coupling in the heterostructures, induced by the magnetostriction of the FeGa layer, in three distinct FeGa nominal thicknesses (50, 100, and 200 nm) (Figure S2a,c,d) by magneto‐optical means (see Experimental Section). The results revealed a clear threshold behavior. At 50 nm, the magnetomechanical related signal was near the detection limit and almost completely undetectable. At this lower thickness, the magnetic volume was insufficient to overcome the mechanical clamping of the stiffer ZnO core, and the film probably suffered from severe discontinuity across the complex 3D topography of the nanosheets. In contrast, a 200 nm FeGa layer yielded only a minimal increase in the optical modulation signal with respect to the 100 nm layer. This indicates a saturation of the effective strain transfer, as thicker films can introduce structural relaxation, higher residual stress, or add rigid dead‐weight that does not further enhance the interface coupling. The added rigidity is also detrimental for the final transferred layers to the elastic PDMS films. These results clearly identified 100 nm as the optimal FeGa thickness that balances high magneto‐mechanical actuation with material efficiency.

Finally, liquid PDMS was spun‐coated to form a layer of ca. 52 µm that conformally covered the ZnO/FeGa nanosheets, which was thermally cured at 80°C for 1 h (Figure 1f; see Experimental Section). After cooling to room temperature, the heterostructure could be cut to the required dimensions and easily peeled off from the Si substrate, leading to the freestanding highly flexible Au/Ti/ZnO/FeGa/PDMS heterostructure (Figure 1g). This PDMS thickness was the thinnest enabling the robust transfer of the Au/Ti/ZnO/FeGa without risk of layer rupture.

### 2.2 Morphological and Structural Characterization

**FIGURE 2:**
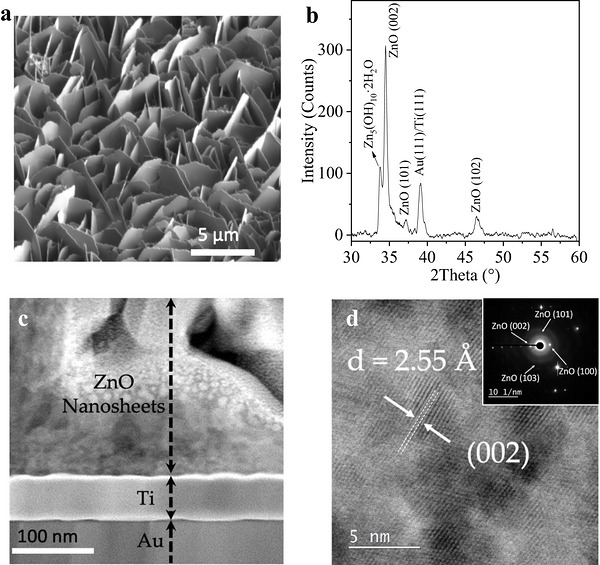
(a) SEM image of the ZnO layer. (b) X‐ray diffraction pattern of the ZnO layer. (c) Low‐resolution and (d) high‐resolution cross‐section TEM images of the FeGa/ZnO layers. The inset in (d) shows the selected area electron diffraction pattern (SAED) of the image.

The scanning electron microscopy (SEM) images of the ZnO layer showed the growth of a highly dense and uniformly distributed network of ZnO nanosheets (Figure 2a) [65]. The X‐ray diffraction (XRD) evidenced that the ZnO layer was hexagonal (hcp) with high crystallinity (Figure 2b), textured along the c‐axis. The XRD also showed the presence of a zinc hydroxide phase, which is likely an intermediate phase in the growth of the hcp‐ZnO phase.

The low‐resolution transmission electron microscopy (TEM) imaging showed a somewhat inhomogeneous ZnO layer (ca. 100 nm) next to the Ti layer, probably due to the growth of the intermediate phases shown in XRD (Figure 2c). After this layer, the ZnO became more homogeneous and branched out into the crystalline nanosheets observed in the SEM (Figure 2c). The interplanar distance in the high‐resolution TEM image and the selective area diffraction confirmed the hcp character of the ZnO layer (Figure 2d).

To confirm the overall structure, energy dispersive x‐ray (EDX) mapping of the TEM cross section of the Au/Ti/ZnO/FeGa composite structure was carried out (Figure S3a). The ZnO nanosheets were estimated to be about 11 nm thick and about 8.5 µm long using the Kramers–Kronig sum method [66] (Figure S3b), in concordance with the SEM images.

Notably, the ZnO nanosheets homogenously incorporated the dissolved Al atoms from the Al seed layer over the whole structure (Figure S3c), thereby forming Al‐doped ZnO nanosheets [62, 63]. Due to the flaky structure of the ZnO, the FeGa layer did not form a completely homogenous coating, but it was preferentially deposited on the top part of the ZnO flakes (Figure S3d). Finally, the thickness of the deposited PDMS elastomer was estimated to be about 52 µm (Figure S4).

This architecture was designed with chemical and mechanical protection in mind. The Au capping electrode layer and the conformal PDMS film act as a robust dual‐barrier system, isolating the active inorganic components from the surrounding environment. This effectively mitigates the risk of corrosion, oxidation of the FeGa layer, or unwanted ion leaching, which are common failure points in bio‐integrated electronics.

### 2.3 Piezoelectric and Magnetic Characterization

**FIGURE 3:**
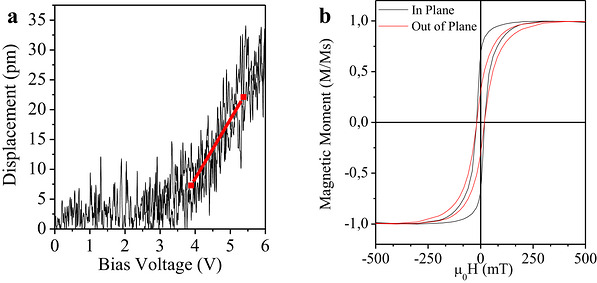
(a) Dependence of the displacement on the bias voltage. The line indicates the linear part of the curve used to extract d_33_. (b) Normalized in‐plane and out‐of‐plane magnetic hysteresis loops of the FeGa/ZnO composite.

The piezoelectric character of the ZnO nanosheets was determined by piezo force microscopy (Figure 3a). From the slope of the displacement curve (see Experimental Section), the estimated piezoelectric coefficient was d_33_ = 11.2 ± 0.3 pm·V^−1^, which is consistent with other ZnO structures [67, 68].

On the other hand, the magnetization measurements of the Au/Ti/ZnO/FeGa/PDMS heterostructures showed that the structure had ferromagnetic behavior with an in‐plane magnetization and a moderate coercivity (μ_0_H_C_ = 20 mT; Figure 3b).

To demonstrate the mechanical effects of the FeGa magnetostriction on the ZnO nanosheets, we performed diffused light scattering measurements under an alternating magnetic (see Experimental Section). Despite the low modulation amplitude (∼200 ppm), the magnetomechanical effect (related to the magnetostriction) was clearly resolved (Figure S2a), confirming that the FeGa deformation was effectively transferred to the ZnO nanosheets to drive the piezoelectric actuation. This signal magnitude strongly suggests that the intrinsic magnetostriction of the sputtered FeGa thin film was high, likely approaching the values of high‐quality bulk Galfenol (300–400 ppm), thereby confirming its high magnetoelastic quality on the 3D ZnO architectures.

Note that bulk magnetoelectric measurements were also attempted. However, the mechanically sensitive and complex nanostructured surface prevented establishing reliable stable contacts for electric measurements without influencing the magnetomechanical properties of the ZnO/FeGa heterostructure.

### 2.4 Flexibility Studies

The highly flexible character of the composite heterostructures was confirmed by measuring the bending angle when applying magnetic fields in different directions using permanent magnets. As can be seen in Figure S5, the heterostructures could easily bend upon the application of magnetic fields. Remarkably, the flaky structure of the ZnO and the discontinuous nature of the FeGa layer allowed maintaining the high flexibility of the PDMS layer without jeopardizing the piezoelectric properties of the ZnO.

### 2.5 Indirect Evaluation of the Magnetically Induced Electric Field Generation

**FIGURE 4:**
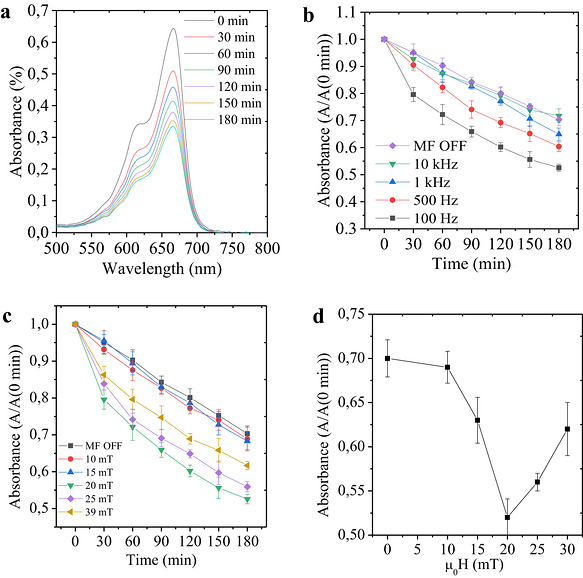
(a) Absorbance spectrum of methylene blue at different reaction times for μ_0_H = 20 mT at 100 Hz. (b) Dependence of the normalized absorbance at 664 nm of the FeGa/ZnO heterostructure on the magnetic field actuation time for different magnetic field frequencies at μ_0_H = 20 mT. (c) Dependence of the normalized absorbance at 664 nm of the FeGa/ZnO heterostructure on the magnetic field actuation time for different magnetic field amplitudes at ω = 100 Hz. (d) Dependence of the normalized absorbance at 180 min on the applied field for ω = 100 Hz. The error bars indicate the standard deviation of the replicated experiments.

In nanostructured samples, the evaluation of the overall generation of electric fields induced by the application of magnetic fields (magnetoelectric effect) is highly complex, due to the difficulty to establish a good electric contact on the grown nanostructured layer. To overcome this limitation, an indirect approach was developed to qualitatively illustrate the electric field generation with alternating magnetic fields. This approach is based on a recent demonstration of the capacity to amplify the activation of peroxymonosulfate (PMS) on metal oxide layers subjected to an alternating potential [69]. The catalytic activation of the PMS generates highly reactive sulfate and hydroxyl radicals that can degrade organic dyes, such as methylene blue, whose degradation can be easily followed by colorimetry. In the present system, the applied magnetic field produces magnetostrictive strain in the FeGa layer, which is transferred to the piezoelectric ZnO. The resulting polarization of ZnO separates electrons and holes, and these charge carriers can transfer into the available electronic states of PMS molecules in solution (HOMO/LUMO), [70] thereby driving their activation. This process leads to the formation of sulfate radicals and other reactive oxygen species that degrade the dye, consistent with previous reports [71]. The amplification of the PMS activation is further reinforced by the negative surface charge of PMS molecules and the generated sulfate radicals, and their dynamic interaction with the induced surface charges at the metal oxide–water interface Therefore, in the case of the ZnO/FeGa structures (before covering with the PDMS elastomer), the metal oxides at their surface can act as catalysts of the PMS activation (Figure S6). This catalytic effect should be amplified by the dynamic surface charges, q(ω), generated by the magnetoelectric actuation induced by an alternating magnetic field H(ω) (Figure S6), thereby accelerating the dye degradation. Since methylene blue changes its color from blue to transparent as the degradation progresses, the reaction can be conveniently tracked by analyzing the evolution of its absorbance spectrum over time (Figure 4a). Consequently, the methylene blue degradation rate provides a qualitative estimation of the electric field generation under various magnetic actuation conditions.

The degradation reaction was first performed under a magnetic field amplitude μ_0_H = 20 mT at varying frequencies. As can be observed in Figure 4a, b, the alternating magnetic field clearly enhanced the methylene blue degradation, reflecting the amplified PMS activation by the induced electric field in the ZnO/FeGa heterostructure. The degradation rate was maximized for lower frequencies, being ω = 100 Hz the most efficient (Figure 4b). Although these effects may suggest that the magnetoelectric coupling response of the heterostructures could be frequency‐dependent, other chemical effects related to the methylene blue degradation (like the inefficient mass transfer toward the surface at high frequencies) may play a more important role. Moreover, mechanical damping in the liquid medium at high frequencies (which may hinder the effective transmission of the mechanical forces between the magnetostrictive and piezoelectric layers) cannot be ruled out.

Next, the catalytic degradation of methylene blue was studied under different magnetic field amplitudes at a fixed frequency of ω = 100 Hz. Interestingly, the effect of the magnetic field strength on the catalytic degradation was not monotonic, showing the largest degradation at 20 mT and 100 Hz. (Figure 4c, d). This effect is likely related to the coercivity of the FeGa layer and the field dependence of the magnetostriction, λ, [72] or the changes in piezomagnetic coefficient, ∂λ/∂H, [73] as the mechanical deformation is mainly triggered when the magnetization is flipped. These results confirm the intense magnetoelectric coupling between the ZnO and FeGa nanostructures and its potential for wireless magnetoelectric stimulation of cells under low frequency and low amplitude magnetic fields.

### 2.6 Cytotoxicity of the FeGa/ZnO Heterostructures

**FIGURE 5:**
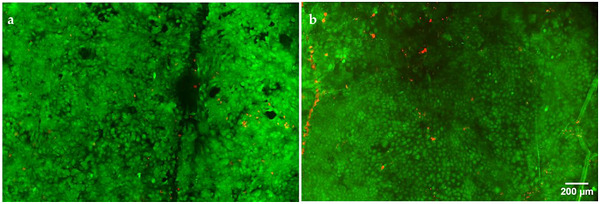
Viability keratinocytes (HaCaT cells) grown on the Au electrode of the flexible Au/ZnO/FeGa/PDMS heterostructures after 7 days of incubation. a) Confocal images of cells seeded on a coverslip (control) and b) on the heterostructures. Live cells are stained with calcein AM (green) and dead cells with ethidium homodimer ‐1 (red).

The observed magnetoelectric coupling motivated the analysis of the magnetoelectric stimulation induced by the flexible Au/ZnO/FeGa/PDMS films on skin cells grown on the Au electrode (see Figure S1). First, to ensure the safety of the flexible magnetoelectric heterostructure, the cytocompatibility of the keratinocyte cell line HaCaT was tested. As expected from the Au biocompatibility, the HaCaT cells could grow on the magnetoelectric heterostructure at high densities, showing no significant differences compared to the glass coverslip control. As can be seen in Figure 5a, b, the cells reached confluence on both the heterostructure and the glass control, indicating that the magnetoelectric material did not negatively affect the cell viability. Although cells directly grew on the Au layer, the magnetoelectric heterostructure was composed of ZnO, FeGa and PDMS. FeGa is a magnetostrictive material that has already been demonstrated to be cytocompatible [74]. For the ZnO nanosheets, the cytocompatibility and immunogenicity of nanostructured ZnO was evaluated on different cell types, including osteoblasts, muscle cells and macrophages. In all cases, ZnO allowed cells to adhere and proliferate without any deleterious effect [65, 75].

**FIGURE 6:**
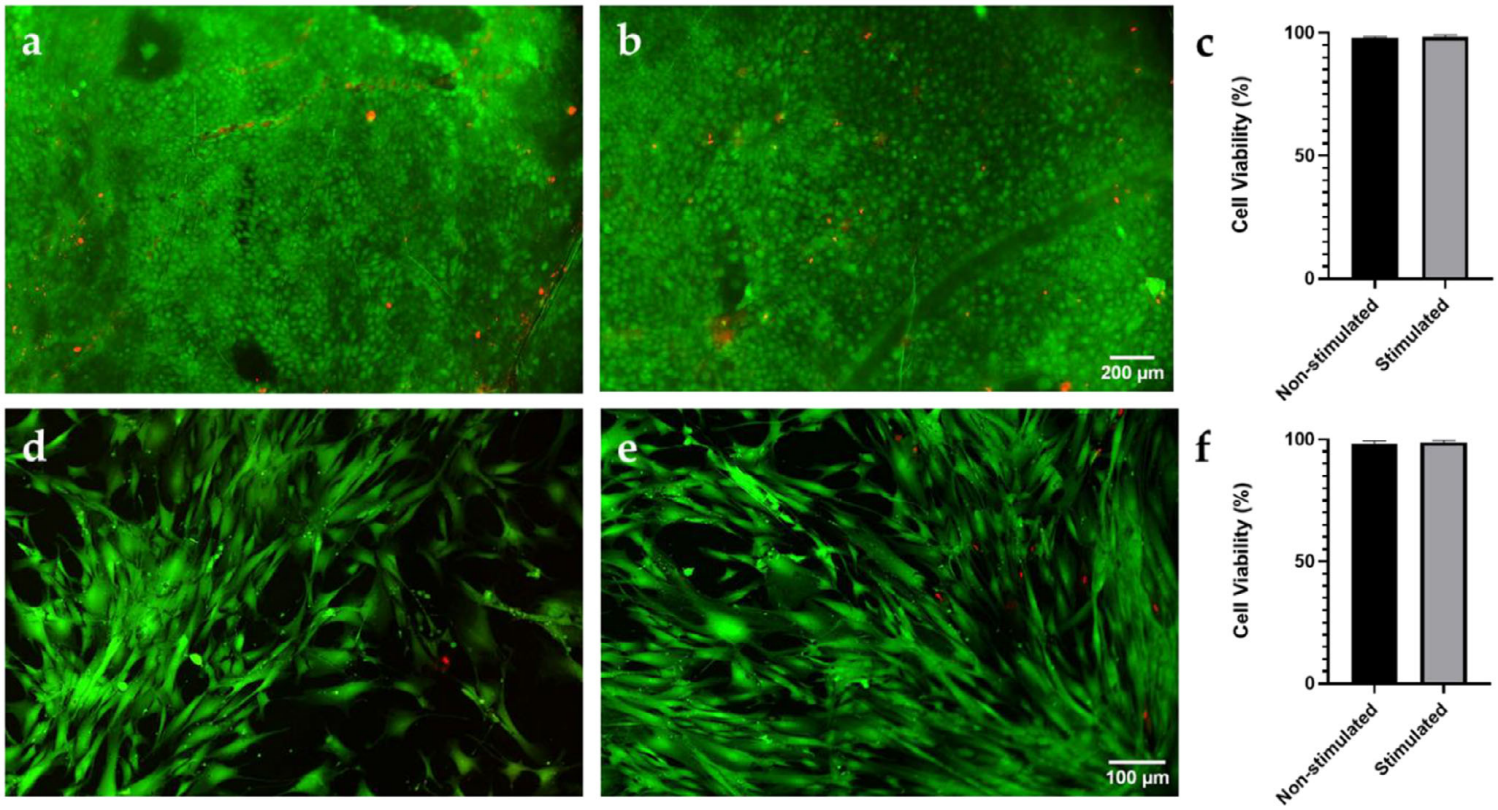
Viability of keratinocytes (HaCaT) and fibroblasts (NHDF) grown on the Au electrode of the flexible Au/ZnO/FeGa/PDMS heterostructures after 7 days with and without magnetic stimulation. The magnetic stimulation was applied 1 h/day. (a) Live/death confocal images of the HaCat cells seeded without stimulation and (b) with 1 h stimulation per day. (c) Percentage of live HaCat cells with/without magnetoelectric stimulation. (d) and (e) Similar confocal images for the NHDF cells. Live cells were stained with calcein AM (green) and dead cells with ethidium homodimer‐1 (red). (f) Percentage of live NHDF cells with and without magnetic stimulation. The data in panels c and f represent the mean ± standard error of the mean.

Next, the safety of the magnetic actuation was studied on HaCaT keratinocytes and NHDF fibroblasts grown on the Au/ZnO/FeGa/PDMS heterostructures under magnetic actuation for 7 days. The results indicated that both HaCaT and NHDF grew on the stimulated material at high densities (Figure 6a–f), showing similar viability as the non‐stimulated ones, with no significant differences (*p* ≤ 0.05). The effect of magnetic stimulation on the cells without the presence of the heterostructures was previously analyzed by our group [22]. The results demonstrated that the magnetic stimulation of 40 mT@100 Hz for 1 h daily had neither detrimental nor beneficial effects on the cells. Moreover, we have also demonstrated the safety of magnetic stimulation when cells were growing on the flexible heterostructure (Figure 6).

It is worth mentioning that, at these stimulation conditions, the magnetic induction heating was negligible, as expected from the low frequency magnetic field used and the low effective thickness of the FeGa layer.

Vibration effects can be also ruled out due to the small mechanical distortions caused by the magnetostrictive actuation (on the order of 10^−4^), being too weak to propagate as macroscopic mechanical vibrations. Furthermore, as the heterostructure is integrated into an elastic PDMS matrix, the nano‐vibrations would be heavily attenuated by the high viscoelastic damping of the elastomer.

### 2.7 Keratinocytes Stratification under Magnetoelectric Stimulation

A healthy epidermis can be considered a stratified tissue composed of basal keratinocytes that express cytokeratin K14 protein. Keratinocytes continuously divide and differentiate, inducing the expression of cytokeratin K10. Once differentiated, keratinocytes are located in the suprabasal layers. However, when the skin is injured, the epidermis and the dermis disappear, requiring a re‐epithelialization process to repair the damaged area. Keratinocytes have the ability to regulate their clonogenic potential and express the differentiated keratin gene (cytokeratin K10), but they initially require reaching cellular confluence in the basal layer [76].

**FIGURE 7:**
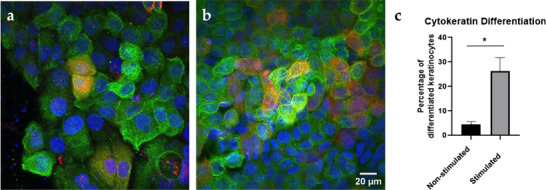
Comparison of the stratification in keratinocytes (HaCaT cells) cultured on the Au electrode of the flexible Au/ZnO/FeGa/PDMS heterostructures for 7 days with and without magnetoelectric stimulation. (a) Immunofluorescence staining of the non‐stimulated cells, and (b) stimulated cells under magnetic actuation. Cytokeratins K14 and K10 protein cells were labelled with anti‐cytokeratin 14 (green), anti‐cytokeratin 10 (red), whereas the nucleus was stained with Hoechst (blue). (c) Percentage of cytokeratin K10 expression in the differentiated HaCaT cells with and without magnetoelectric stimulation. The data represent the mean ± standard error of the mean, ^*^ *p* < 0.05.

To determine whether the magnetoelectric actuation influences keratinocyte differentiation, a stratification assay was performed on HaCaT cells (Figure 7). This assay clearly showed that the magnetoelectric stimulation for 7 days (1 h per day) induced a remarkable 5‐fold increase in the appearance of keratinocytes with a differentiated phenotype expressing K10, compared to cells incubated without magnetic actuation. Therefore, the generated electric field was able to increase the metabolic activity and, consequently, the proliferation of keratinocytes. Hence, the increased proliferation of keratinocytes allowed the cells to reach confluence earlier, which in turn enabled their differentiation and stratification. Proliferation is essential to close the wound and start the stratification of the epithelial cells in order to reestablish the skin barrier function and transepithelial potential. The effect of the electric fields on epithelial was demonstrated earlier in several studies [40, 77]. Our results indicate that the electrical stimulation generated under magnetic field is able to enhance the stratification of keratinocytes directly grown onto the heterostructures.

### 2.8 Collagen Production by Fibroblasts under Magnetoelectric Actuation

Under the epidermis, an extracellular matrix (ECM) primarily composed of type I and III collagen fibers is found. This collagen is produced, secreted, and organized by fibroblasts, and is essential for regulating cell growth and survival in the dermis, [78] being also one of the essential mechanisms in wound healing. Therefore, an increased collagen production translates to an enhanced capacity for epithelial wound regeneration [79].

**FIGURE 8:**
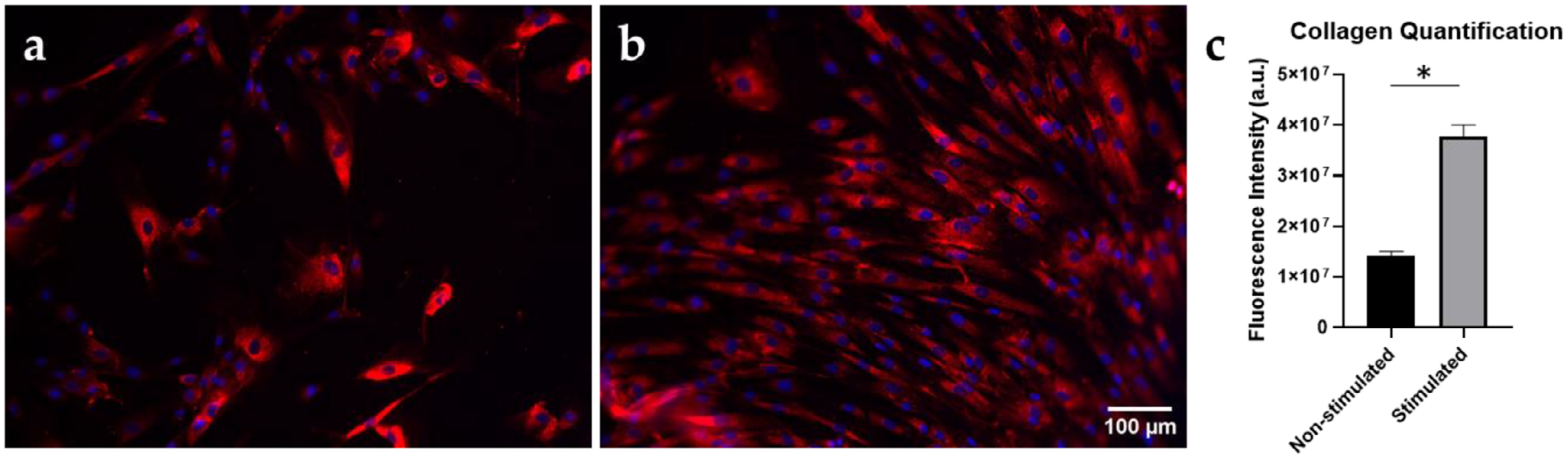
Comparison of collagen formation in fibroblasts (NHDF Cells) cultured on the Au electrode of the flexible Au/ZnO/FeGa/PDMS heterostructures for 7 days. (a) Immunofluorescence of collagen in non‐stimulated NHDF cells and (b) under magnetic actuation for 7 days (1 h stimulation per day). The cells were stained with anti‐collagen type I (red) and the nucleus was stained with Hoechst (blue). (c) Dependence of the integrated fluorescence intensity of the NHdF cells per image in the two conditions. The data represent the mean ± standard error of the mean, ^*^ *p* < 0.05.

To determine the effect of magnetoelectric stimulation on collagen production, an assay was conducted on fibroblast cells. After being exposed to magnetic actuation for 1 h daily over seven days, the cultures showed a remarkable 3‐fold increase in type I collagen production compared to non‐stimulated cells (Figure 8). These findings suggest that magnetoelectric stimulation promotes fibroblasts proliferation and type I collagen production. Since collagen is the main component of the extracellular matrix of the dermis, the increase in its production can influence directly the wound healing and tissue regeneration. According to other authors, increases in type I collagen production in fibroblasts can also be obtained after applying pulsed electrical stimulation [80]. Thus, our results are in accordance with previous studies that indicated the potential of electrical stimulation to accelerate wound healing.

### 2.9 Wound Healing Assays

In epithelial wound healing, fibroblasts migrate from the healthy tissue to the damaged area, promoting wound contraction, ECM deposition, and tissue reorganization to facilitate the closure of the injured area [81].

**FIGURE 9:**
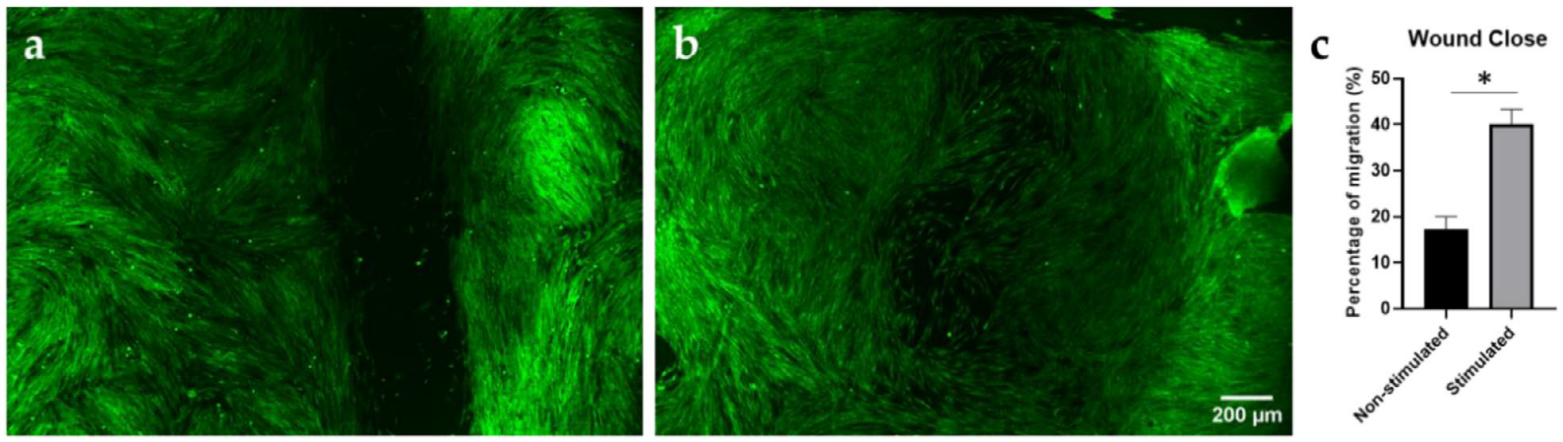
Fibroblast (NHDF cells) migration on the Au electrode of the flexible Au/ZnO/FeGa/PDMS heterostructures determined by wound healing assay. Immunofluorescence of NHDF cells incubated (a) without stimulation and (b) 24 h after 1 h of magnetic actuation. The cells were stained with CellTacker CMFDA (green). (c) Percentage of migration of the NHDF cells in the two conditions. The data represent the mean ± standard error of the mean, ^*^ *p* < 0.05.

To evaluate the effects of magnetoelectric stimulation on fibroblast migration, a wound healing assay was performed on NHDF cells over 24 h after 1 h of stimulation. The results (Figure 9) show a dramatic increase in the percentage of area covered by fibroblasts (more than double) under magnetic actuation compared to the non‐stimulated cells. These findings suggest that the magnetically actuated flexible Au/ZnO/FeGa/PDMS heterostructures significantly enhance cell migration. The effect of electric fields in cell migration has been widely studied. Indeed, electrotaxis is the directed cell migration in response to an electric field [77]. This effect, when combined with the previously observed increase in type I collagen production, indicates that this technology could be key to developing novel wirelessly controlled active dressings for improving wound healing processes.

Magnetoelectric heterostructures present a robust and innovative platform for accelerating the healing of chronic wounds. Acting as multifunctional wound dressings, they combine physical protection against bacterial contamination with the capacity to deliver localized magnetoelectric stimulation that supports tissue regeneration. Their highly flexible components allow the heterostructures to conform to the skin morphology (Figure S7). In addition, the magnetoelectric response can be activated wirelessly by applying an external magnetic field, enabling minimally invasive and spatially controlled stimulation at the wound site. Together, these attributes position magnetoelectric heterostructures as a technologically advanced and clinically relevant alternative that may overcome several limitations of current chronic wound therapies

Notably, the magnetoelectric actuation could also provide an indirect therapeutic benefit in infected wounds, as localized electric stimulation has been shown to modulate and stimulate immune cell activity, which can enhance the tissue's natural defense mechanisms to fight off bacterial pathogens and biofilm formation in infected wounds [82]. In addition, the device architecture is highly versatile and could be readily adapted for active infection control. For instance, replacing the current Au electrode with inherently antimicrobial materials such as Ag, Cu, or their alloys, would seamlessly integrate direct antibacterial functionality into the platform without compromising the underlying magnetostrictive and piezoelectric mechanisms.

## 3 Conclusion

Highly flexible and conformable Au/ZnO/FeGa/PDMS magnetoelectric heterostructures have been developed. The heterostructures exhibited piezoelectric and ferromagnetic behavior and the coupling between both effects enabled generating electric fields under magnetic actuation. The indirect magnetoelectric analysis via PMS activation, showed that the most efficient magnetic field amplitude was 20 mT, which was correlated to the coercivity of the FeGa layer. The flexible heterostructures showed high potential for magnetoelectric stimulation of skin cells. The material exhibited a low cytotoxicity, with high cell viability and proliferation. The skin related assays demonstrated a remarkable improvement of the keratinocytes (HaCaT) stratification, as well as enhanced type I collagen production and cell migration of fibroblasts (NHDF) under magnetoelectric stimulation with low amplitude and low frequency magnetic fields. This study demonstrates that the Au/ZnO/FeGa/PDMS heterostructures, under magnetic actuation, can offer an effective and non‐invasive strategy for skin wound regeneration. The scalability and biocompatibility of the magnetoelectric material, along with the outstanding improvement in skin cells differentiation and migration, underscores the potential of this technology as magnetoelectric dressings for clinical applications. Translating this in vitro proof‐of‐concept to a clinical setting will require transitioning through the pre‐clinical and regulatory pathway. First, the fabrication of the flexible ZnO/FeGa/PDMS heterostructure should be scaled while ensuring the long‐term integrity of the biocompatible encapsulation under physiological conditions. Second, rigorous in vivo studies using animal wound models are essential to evaluate the systemic biocompatibility and the real‐world efficacy of magnetoelectric stimulation on accelerated tissue regeneration. Finally, the system would need to be integrated with a portable magnetic actuator (Figure S7) to allow efficient actuation in clinical settings, followed by phased human clinical trials to meet regulatory safety and performance standards for medical devices.

## 4 Experimental Section

### 4.1 Sample Fabrication

Low‐resistivity p‐doped silicon wafers (<0.025Ω · *cm*) were cut into pieces of approximately 30 × 20 mm^2^ and cleaned using oxygen plasma (PVA TePla Inc., PS210) for 5 min to remove organic contaminants and improve surface wettability. The substrates were then loaded into an electron‐beam evaporator (AJA International Inc., ATC‐8E Orion), where the metal electrode and seed tri‐layer (Au/Ti/Al) was deposited at a rate of 0.05 *nm*/*s* in the following order: Au (50 nm) / Ti (10 nm) / Al (100 nm).

For the hydrothermal growth of ZnO, an equimolar aqueous solution (5 mm) was prepared by dissolving 74.37 mg (0.25 mmol) of zinc nitrate hexahydrate (*Zn*(*NO*_3_)_2_ · 6*H*_2_ *O*) (Sigma) and 35.05 mg (0.25 mmol) of hexamethylenetetramine (HMTA) (Sigma) in 50 mL of Milli‐Q water. The solution was first sonicated for 5 min for dissolving (*Zn*(*NO*_3_)_2_ · 6*H*_2_ *O*), followed by the addition of HMTA and an additional 5 min of sonication, and finally magnetically stirred for 10 min to ensure complete homogenization. The resulting solution was transferred into hermetically sealable containers, and the substrates were placed upside down floating at the liquid/air interface of the solution with the metal‐coated side facing the liquid, ensuring that they remained floating without mechanical damage. The containers were sealed and placed in a convection oven preheated to 80°C, where the reaction was maintained for 14 h. After the synthesis, the samples were removed, allowed to cool down, and immediately immersed in ethanol for 5–10 s to remove residual species and to minimize capillary forces during drying. The samples were then placed on a clean, lint‐free surface, covered with an inverted glass container to prevent dust contamination, and left to dry overnight under ambient conditions.

The FeGa layer was deposited on top of the ZnO nanosheets by sputtering (ATC Orion 5 from AJA International, 100 W DC, deposition rate 7.95 nm/min) using a Fe_80_Ga_20_ target. The composition of the layers was confirmed to be Fe_80_Ga_20_ by EDX.

For encapsulation and release, the PDMS base polymer and the curing agent (SYLGARD 184 Silicone Elastomer Kit) were mixed at a 10:1 ratio, thoroughly stirred, and degassed under low vacuum for 15 min until all visible bubbles were removed. The mixture was then spin–coated onto the sample at 1000 rpm for 60 s, yielding a PDMS layer of approximately 52 µm thickness, followed by thermal curing at 80°C for 1 h. After cooling to room temperature, the sample was cut to the desired dimensions and carefully peeled off from the Si substrate, resulting in a freestanding flexible heterostructure with the configuration Au/Ti/ ZnO/Fe_80_G_20_/PDMS.

### 4.2 Surface Morphology

The surface morphology of the composite was investigated by SEM using a Quanta 650 FEG SEM, operated at 10 kV.

### 4.3 X‐Ray Diffraction (XRD)

The XRD analysis was performed using Malvern Panalytical X'pert Pro MRD using Cu Κ_α_ radiation (Κ_α_ = 1.54187 Å).

### 4.4 Transmission Electron Microscopy

TEM and high‐resolution TEM (HR‐TEM) were performed using a TEM/STEM Jeol ARM 200 operated at 80 kV and a FEI Tecnai G2 F20 with a 200 kV field emission gun (FEG). The energy dispersive x‐ray analysis was carried out in the TEM/STEM Jeol ARM 200 CF STEM equipped with Jeol Centurio EDXS system and a 100 mm^2^ SDD detector. To examine the cross‐sections of the nanostructures, lamellas of the samples were prepared by focused ion beam (FIB) milling using a gallium ion source (FEI Nanolab Helios 650 ‐ FIB).

Note that TEM imaging (Figure 2 and Figure S3) confirmed a sharp interface between the diverse layers.

### 4.5 Magnetic Properties

The magnetic properties were evaluated at room temperature using a MicroSense (LOT‐Quantum Design) vibrating sample magnetometer with a maximum applied field of 2 T, applied either in‐plane or out‐of‐plane with respect to the substrate.

### 4.6 Magnetomecahnical Coupling

To demonstrate magnetomechanical effects due to the magnetostriction of the sputtered FeGa and its mechanical coupling with the ZnO nanosheets, we have carried out diffused light scattering measurements under an alternating magnetic field of µ_0_H = 25 mT amplitude at a frequency of ω = 277 Hz. To avoid the generation of conventional magneto‐optic Kerr effect (MOKE) modulation, we used a transversal configuration (i.e., magnetization perpendicular to the light incidence plane) and s‐polarized (TE) laser light (808 nm) with an angle of incidence of 45°. The scattered light was collected by an optical fiber bundle perpendicular to the sample surface and separated ca. 20 mm from it. The collected light was detected by an amplified photodiode and analyzed by a Moku:Go oscilloscope. In this configuration, any displacement of the ZnO/FeGa caused by the magnetrostriction when the magnetic field is modulated, should cause a small modulation of the scattered intensity collected by the fiber cores, acting as pinholes. Importantly, this effect should be independent of the sign of the magnetic field, consequently the signal should have a 2ω frequency (where ω is the frequency of the magnetic field). As can be observed in Figure S2a, a 2ω optical signal (related to magnetostriction) could be clearly observed, despite the small intensity variations, thus demonstrating that the FeGa magnetostriction was able to generate a mechanical deformation in the ZnO nanosheets, which would translate in a piezoelectric actuation. As comparison, we include the optical signal observed in the p‐polarized light, which yields a MOKE intensity modulation that, as expected, depends on the direction of the magnetic field (and, thus, it exhibits a 1ω signal dependence), showing the hysteresis loop of the FeGa layer (Figure S2b).

### 4.7 Piezoelectricity

The piezoelectricity was locally probed by piezo‐response force microscopy (PFM), using an Asylum MFP‐3D Classic AFM and a NT‐MDT NTEGRA Prima AFM. For the determination of the d_33_ piezoelectric coefficient, a PtIr5‐coated tip and a cantilever with a spring constant of k = 2.8 N·m^−1^ was used. The coefficient was estimated by applying +6 V_DC_, plotting mechanical deformation vs. applied voltage, and calculating the slope of the linear part (Figure 3), which is equal to the d_33_ piezoelectric coefficient. The experiment was repeated 3 times in different locations and the d_33_ values are given as average ± standard deviation of these measurements. Note that the non‐linear behavior of the displacement curve in Figure 3a is probably due to the semiconducting and metallic character of the ZnO layer and PFM tip, respectively, which form a Schottky barrier [83].

### 4.8 Magneto‐Mechanical Bending Tests

Cantilevers composed of Au/ZnO/FeGa/PDMS heterostructures (1 × 5 mm^2^) were produced by laser cutting. The cantilevers were clamped on one side and subject to the magnetic field gradient generated by a 12 mm diameter spherical FeNdB permanent magnet, which was approached toward the heterostructures from different directions and orientations.

### 4.9 Indirect Evaluation of the Overall Magnetoelectric‐Induced Electric Fields by the Catalytic Degradation of Methylene Blue

The magnetic field‐induced magnetoelectric response of the structures was evaluated by observing their catalytic effect in the methylene blue degradation induced by the catalytic activation of peroxymonosulfate (PMS) under alternating electric fields. Although this method is not quantitative, changes in degradation (and thus the global electric field generation) can be compared between different conditions. To initiate the degradation procedure, methylene blue was mixed with PMS at 0.3 mm. The ZnO/FeGa heterostructure grown on silicon substrate (10 mm x 10 mm) was immersed in 2 mL of the methylene blue‐PMS solution in a quartz cuvette and placed inside a ferrite electromagnet with magnetic fields of varying strengths (from 0 mT to 30 mT) and frequencies (from 0 Hz to 10 kHz). The samples were oriented in‐plane to the applied field, and the experiment was conducted at room temperature. The degradation was monitored by measuring in situ the absorbance spectrum of methylene blue at 30 min intervals for 180 mins using a Flame‐S‐UV–vis miniature spectrometer. The experiments were done in triplicate.

### 4.10 Cell Cultures

The cell cultures of a finite cell line of normal human dermal fibroblasts (NHDF; ATCC) and an immortalized keratinocytes cell line (HaCaT; CLS) were cultured under standard laboratory conditions (37°C and 5% CO_2_) to ensure the reproducibility and cellular viability of the experiments. Both cell lines were cultured in Dulbecco's Modified Eagle Medium (DMEM; ThermoFisher Scientific) supplemented with 10% fetal bovine serum (FBS; ThermoFisher Scientific). The cells were sub‐cultured before they reached approximately 95% confluence.

### 4.11 Sterilization and Set Up of the Au/ZnO/FeGa/PDMS Heterostructures

A sterilization lamp (59s, Health Live Innovation), with a validated efficacy of 99.9%, was used to sterilize the Au/ZnO/FeGa/PDMS heterostructures. Additionally, the heterostructures were adhered on 35 mm diameter Petri dishes using sterilized silicone to prevent buoyancy and to ensure complete coverage by the culture medium.

### 4.12 Cytotoxicity Assays

To evaluate the cytotoxicity of the heterostructures, the viability of HaCaT cells in direct contact with the Au top layer of the Au/ZnO/FeGa/PDMS heterostructures was measured. For this purpose, 150 000 cells were seeded on the Au surfaces of the heterostructures, which were placed inside 35 mm plates as described in the previous section. In addition, glass coverslips were used as controls. The plates were incubated under standard conditions and analyzed 7 days after seeding. The medium was changed every 3–4 days.

The same test was performed on HaCaT and NHDF cells under magnetic actuation. Cells were seeded as described in the previous section. After 24 h, the samples with the cells adhered on the Au electrode were transferred to sterile cuvettes and 2 mL of culture medium with 1% antimycotic and antibiotic was added. The cuvettes were covered with sterile parafilm and placed in the magnetic actuation system, and were actuated under an alternating magnetic field of 40 mT at 100 Hz for 1 h per day over seven days.

The viability was evaluated using the LIVE/DEAD cytotoxicity/viability kit from Invitrogen (Thermo Fisher Scientific), which stains live cells in green with calcein AM and dead cells in red with ethidium homodimer‐1.

The stimulated or non‐stimulated cells grown on Au top layer of the Au/ZnO/FeGa/PDMS heterostructures were analyzed under a fluorescence microscope (IX71, Olympus). The experiments were done in duplicate.

### 4.13 Stratification Assays

To assess the stratification capability of the HaCaT cells, a double immunodetection of K14 and K10 was performed on the stimulated and non‐stimulated samples. 150 000 cells were seeded on the Au surface of the Au/ZnO/FeGa/PDMS heterostructures following the methodology described above. Subsequently, the seeded samples were incubated for 24 h at 37°C and 5% CO_2_, and then transferred to the magnetic actuation system, where they were stimulated under the parameters described above.

Seven days after the first actuation, immunodetection of K10 and K14 was conducted. For this purpose, the cell medium was removed from the wells containing the heterostructures, washed with PBS, and the cells were fixed with 4% paraformaldehyde (PFA) for 20 min. Then, they were permeabilized with 0.5% PBS‐triton and 1% PBS‐tween for 30 min each. Subsequently, the cells were blocked with 1% PBS‐BSA for 20 min.

For dual immunodetection, 100 µL of primary antibody anti‐cytokeratin 10 (EP1607ICHCY, Abcam), diluted to a concentration of 1:200, were applied to a piece of parafilm. The material was then placed upside down onto the antibody drop. After 1.5 h, a wash with PBS‐BSA was performed, and the same process was repeated with the primary antibody anti‐cytokeratin 14 (LL002, Abcam), diluted to a concentration of 1:200. Next, another wash with PBS‐BSA was carried out, and 100 µL of secondary antibodies at a concentration of 1:400 was added: AlexaFluor594 goat anti‐rabbit (A‐11037, ThermoFisher Scientific) for K10 and AlexaFluor488 anti‐mouse Ig (A‐10680, ThermoFisher Scientific) for K14, along with Hoechst at a concentration of 1:1000, which gives blue fluorescence of nuclei. After an additional 1.5 h, a final wash with PBS was performed.

The materials were analyzed under a fluorescence microscope (IX71, Olympus) and a confocal laser scanning microscopy (CLSM; LSM 980, Zeiss). The experiments were done in duplicate.

### 4.14 Collagen Production Assays

The differentiation capacity of NHDF cells under magnetoelectric stimulation was evaluated by immunodetection of type I collagen. For this purpose, 150 000 cells were seeded onto the Au electrode of the Au/ZnO/FeGa/PDMS heterostructure, following the methodology described above. Subsequently, they were incubated for 24 h at 37°C and 5% CO_2_, and then transferred to the magnetic actuation system, where they were actuated 1 h per day with magnetic fields of either 20 mT or 40 mT at a frequency of 100 Hz.

After 7 days, the cells on the heterostructures were washed, fixed, permeabilized, and blocked as described above. For immunodetection, 100 µL droplet of primary anti‐type I collagen antibody (EPR7785, Abcam) at a concentration of 1:200 was applied. The material was then placed facing down on the droplet and incubated for 1.5 h at room temperature. Subsequently, the samples were washed with PBS‐BSA and 100 µL of secondary antibody, AlexaFluor594 anti‐Rabbit (A‐11012, ThermoFisher Scientific), at a concentration of 1:400, along with Hoechst at a concentration of 1:1000, were added. This was incubated for 1 h and a half, followed by washing with PBS.

The materials and controls were analyzed under a fluorescence microscope (IX71, Olympus) and a CLSM (LSM 980, Zeiss). The quantification was performed using Image J (NIH software) and collagen quantification was displayed as fluorescence intensity per field. The experiments were done in duplicate.

### 4.15 Cell Migration Assays

To analyze cell migration, a wound healing assay was performed. Initially, the Au/ZnO/FeGa/PDMS heterostructure was prepared as described above. Subsequently, a silicone insert with two wells and a cell‐free central gap of 500 µm (80209, Ibidi) was placed on the Au electrode of the heterostructure. Both wells were filled with 70 µL of culture medium containing 25 000 NHDF cells and incubated for 24 h at standard conditions.

After 24 h, the insert was removed, the cells were stained with green fluorescence CellTracker CMFDA (C2925, ThermoFisher Scientific), and the Au/ZnO/FeGa/PDMS heterostructure containing the cells was transferred to the magnetic actuation system and stimulated for 1 h using the parameters described above.

The fibroblasts were grown on the Au electrode over 24 h and the controls were analyzed under a fluorescence microscope (IX71, Olympus). The experiments were done in duplicate.

### 4.16 Statistical Analysis

To evaluate the distribution of the data obtained in each assay, a normality test with a significance level of 0.05 was initially applied. For analyzing cell viability data, Fisher's exact test was used, and the percentage of live cells relative to the total was represented. For data related to the HaCaT stratification, collagen production and migration of NHDF cells, an independent samples T‐test was conducted to compare the means between groups. The quantitative data was presented as the mean ± standard error of the mean. All the biological experiments were done in duplicate. The statistical analyses were performed using GraphPad Prism 8 (GraphPad Software, San Diego, CA, USA). A *p*‐value of *p* ≤ 0.05 was considered statistically significant, where ^*^ represents significances below 0.05.

## Conflicts of Interest

The authors declare no conflict of interest.

## Supporting information




**Supporting File**: advs76041‐sup‐0001‐SuppMat.docx.

## Data Availability

The data that support the findings of this study are available from the corresponding author upon reasonable request.
