# Supplementary material for: Highly Flexible and Conformable ZnO/FeGa Magnetoelectric Heterostructures for Skin wound Healing
Source: Adv Sci (Weinh). 2026 Jun 12:e23781. Online ahead of print. doi: 10.1002/advs.202523781 (PMC13337036; doi:10.1002/advs.202523781)
Supplement: Supplementary file 1 — Supporting File: advs76041‐sup‐0001‐SuppMat.docx. [file ADVS-9999-e23781-s001.docx]

**Supporting Information**

**Highly flexible and conformable ZnO/FeGa magnetoelectric heterostructures for skin wound healing**

Filippos Perdikos,*^#^* Laia Bagur,*^#^* Cristina Vaca, Aritz Lafuente, Joaquin Llacer-Wintle, Bojan Ambrožič, Christina Stefani, Minsoo Kim, Goran Dražić, Darja Lisjak, Jordi Sort, Xiang-Zhong Chen, Maria José Esplandiu, Salvador Pané,^*^ Carme Nogués, Josep Nogués, Andreu Blanquer,^*^ Borja Sepúlveda^*^

**Figure S1.** Schematic of the fabrication process including: deposition of the Au/Ti/Al layers by ebeam evaporation, hydrothermal growth of the Zn(Al)O nanoflakes, deposition of the FeGa layer by sputtering, deposition of the PDMS film by spin coating, and final released the elastic Au/ZnO/FeGa/PDMS heterostructures with skin cells growth on the Au electrode.

|  |  |
| --- | --- |
|  |  |

**Figure S2**. a) Relative variations of the scattered optical signal, ΔS_s_/S_s_, of the ZnO/FeGa nanosheets (with a 100 nm thick FeGa layer), for an s-polarized light incident at 45º, when the alternating magnetic field at a frequency of ω = 277 Hz was applied perpendicular to the incident plane of the light. b) Relative variations of the scattered signal for the p-polarized light, ΔS_p_/S_p_, under identical magnetic conditions showing the magneto-optic Kerr effect (MOKE). c) and d) Relative variations of the scattered signal of the ZnO/FeGa nanosheets, for an s-polarized light incident at 45º when the FeGa layer nominal thickness was 200 nm and 50 nm, respectively.


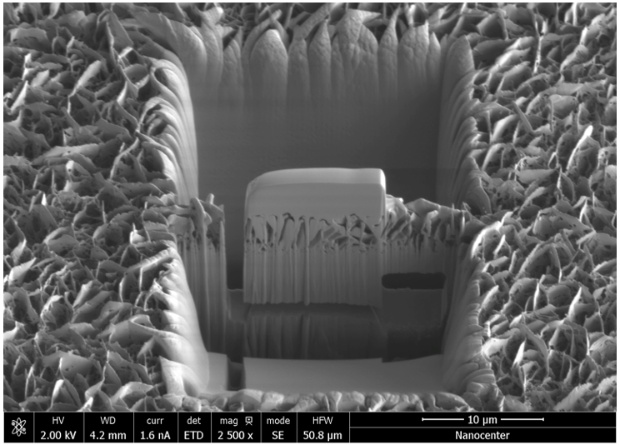


a


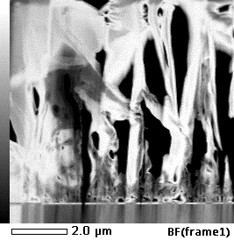


c

b

|  | c |
| --- | --- |
|  | 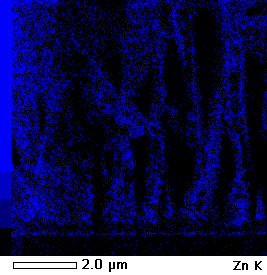 |


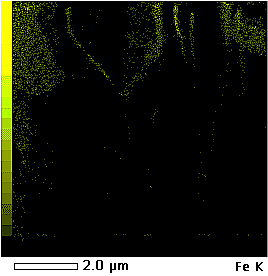


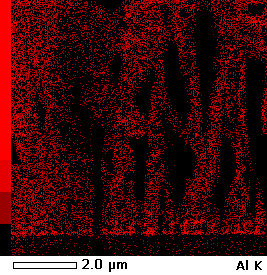


e

d

**Figure S3.** (a) Tilted SEM image of the lamella preparation of the FeGa/ZnO structures using FIB. (b) SEM image and EDX elemental mapping of the ZnO/FeGa structures using the (c) Zn; (d) Al and (e) Fe edges, respectively.

**Figure S4.** SEM cross-section of the Au/Ti/ZnO/FeGa/PDMS heterostructures.

|   b 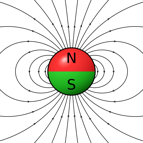 movement  movement 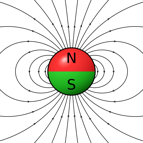 |
| --- |
| 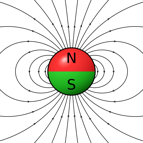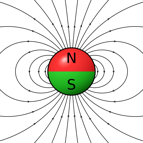   c  movement  movement |
| 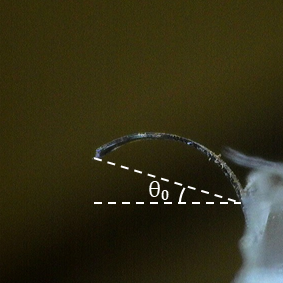 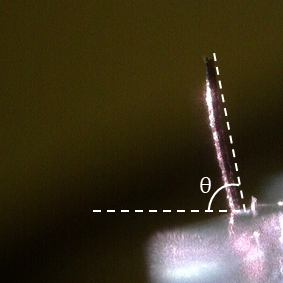 |

**Figure S5.** Magnetomechanical response of the FeGa/ZnO composite using a magnet moving (a) perpendicular (H_n_) and (b) parallel (H_p_) to the Au/Ti/ZnO/FeGa/PDMS cantilevers clamped on one side; (c) The clamped cantilever with the initial angle defined (θ₀) without magnetic field (left); and the resulting angle of the cantilever (θ) when the magnetic field is applied (right).

a

Si substrate

Au

ZnO/FeGa

**Figure S6.** Schematic of the experimental configuration to indirectly show the magnetoelectric coupling at the FeGa heterostructures induced by an alternating magnetic field H(ω) generating dynamic surface charges q(ω) at the ZnO/FeGa-water interface, to amplify the catalytic activation of PMS.


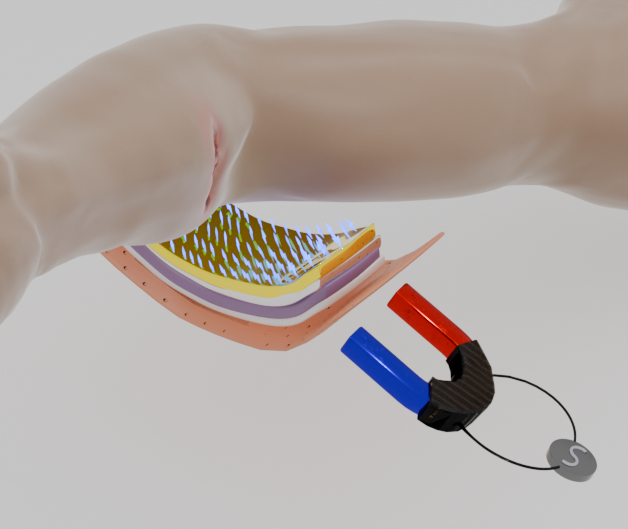


**Figure S7.** Illustrative figure depicting the possible application of the magnetoelectric heterostructures in clinical wound healing.
